# Supplementary material for: Accelerating flash droughts induced by the joint influence of soil moisture depletion and atmospheric aridity
Source: Nat Commun. 2022 Mar 3;13:1139. doi: 10.1038/s41467-022-28752-4 (PMC8894453; doi:10.1038/s41467-022-28752-4)
Supplement: Supplementary file 1 — Supplementary Information [file 41467_2022_28752_MOESM1_ESM.pdf]

Supplementary Materials for

Accelerating flash droughts induced by the joint influence of soil moisture depletion and atmospheric aridity

Yamin Qing[1,†], Shuo Wang[1,2,*,†], Brian C. Ancell[3] and Zong-Liang Yang[4]

1. Department of Land Surveying and Geo-Informatics and Research Institute for Land and Space, The Hong Kong Polytechnic University, Hong Kong, China
2. Shenzhen Research Institute, The Hong Kong Polytechnic University, Shenzhen, China
3. Department of Geosciences, Texas Tech University, Lubbock, TX, USA
4. Department of Geological Sciences, The John A. and Katherine G. Jackson School of Geosciences, University of Texas at Austin, Austin, TX, USA

*Corresponding author: Shuo Wang (shuo.s.wang@polyu.edu.hk)
[†]These authors contributed equally to this work.

This file includes:

Supplementary Text
Supplementary Figs. 1 to 19
Supplementary Table 1

Supplementary Text

GLEAM dataset

The Global Land Evaporation Amsterdam Model (GLEAM) version 3.1a provides observationally constrained global daily surface (0–10 cm) and root-zone (10–250 cm) SM spanning the period from 2000 to 2020. The GLEAM surface SM was produced by employing an improved SM data assimilation system using three independent SM datasets, including two satellite-based SM products from the European Space Agency (ESA) Climate Change Initiative (CCI) and Soil Moisture Ocean Salinity (SMOS) and the surface SM from the Noah model in the Global Land Data Assimilation System (GLDAS). The root-zone SM was modelled from a three-layer water balance module with the input of precipitation infiltration and the outputs of evapotranspiration and drainage. The forcing data of the water-balance module includes Multi-Source Weighted Ensemble Precipitation (MSWEP) precipitation, ERA-Interim radiation and air temperature, CCI-LPRM vegetation optical depth, ESA CCI and GLDAS-Noah SM, etc.

GLDAS-2 dataset

NASA GLDAS-2 (Global Land Data Assimilation System Version 2) models include VIC, Noah, and Catchment land surface models (CLSM) with a spatial resolution of 1.0 degree and a temporal resolution of three hours for 2000−2020. The goal of the GLDAS is to ingest satellite- and ground-based observational data products, using advanced land surface modeling and data assimilation techniques, in order to generate optimal fields of land surface states and fluxes. Daily surface (0−10 cm) and root-zone (0−200 cm) SM were obtained from VIC, Noah, and CLSM.

In this study, we compared the annual total SM as well as the 40th- and 20th-percentile SM from GLEAM and GLDAS, and we find that there is a considerable difference in the patterns of the VIC model and other three datasets, especially for the patterns of 40th- and 20th-percentile SM (Fig. 1). Thus, we selected the SM data from GLEAM, Noah, and CLM datasets. To further access the performance of these three datasets on capturing flash drought events. We compared flash drought frequencies identified by three different datasets. We find that even though the spatial patterns of flash drought frequencies detected based on three datasets show some difference, hot spots are consistent and the difference in the total number of flash droughts identified from three datasets lies between 0–7 (Fig. 2). Therefore, we used these three datasets to conduct a robust assessment of flash droughts all over the world.

Climatic data used for calculating flash droughts based on the duration-based definition

The daily actual evaporation (often referred to as “evapotranspiration”) (ET) obtained from Multi-Source Weighted Ensemble Precipitation (MSWEP) (http://www.gloh2o.org/mswep/), together with near-surface T and P from the Modern-Era Retrospective analysis for Research and Applications, Version 2 (MERRA-2), was used to identify flash drought events based on the duration-based definition proposed by Mo and Lettenmaier (2015, 2016) (https://gmao.gsfc.nasa.gov/pubs/docs/McCarty885.pdf).

Supplementary Figures

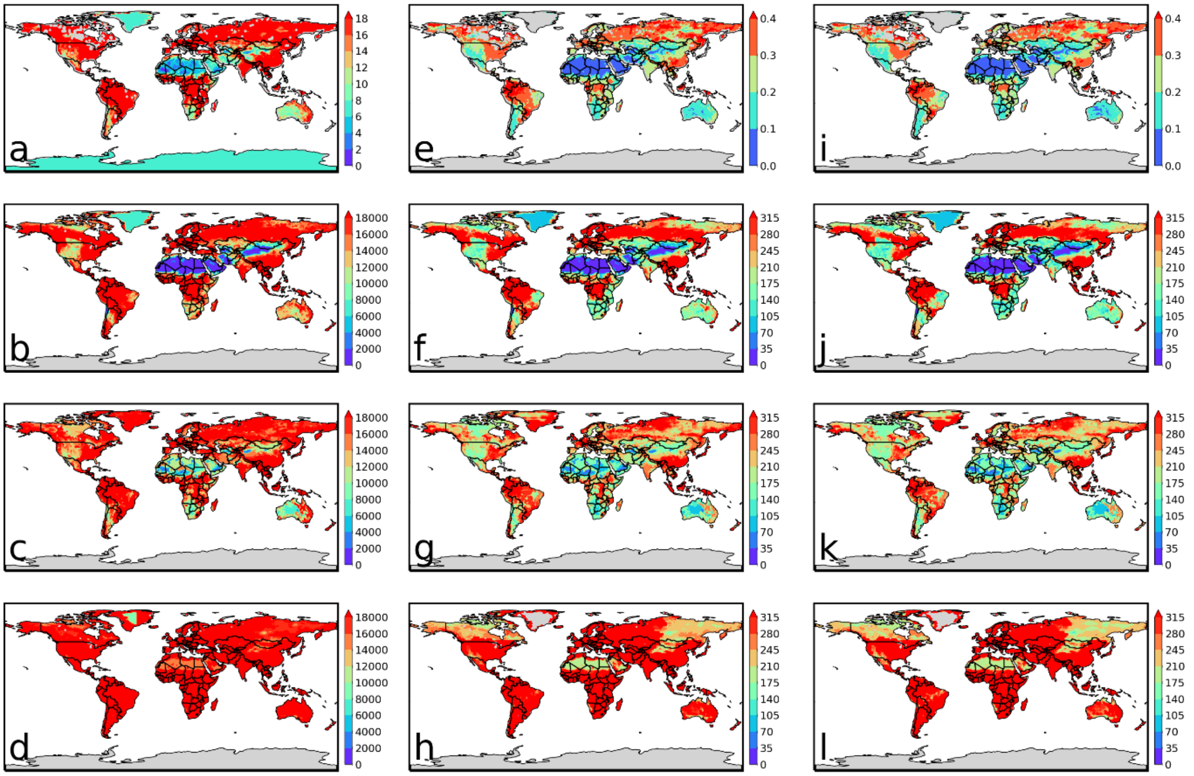

Supplementary Fig. 1 Comparison of SM from four datasets. a−d Spatial pattern of annual total SM for GLEAM, Noah, CLSM, and VIC datasets. **e−h** Same as **a−d**, but for the 40th percentile SM. **i−l** Same as **a−d**, but for the 20th percentile SM.

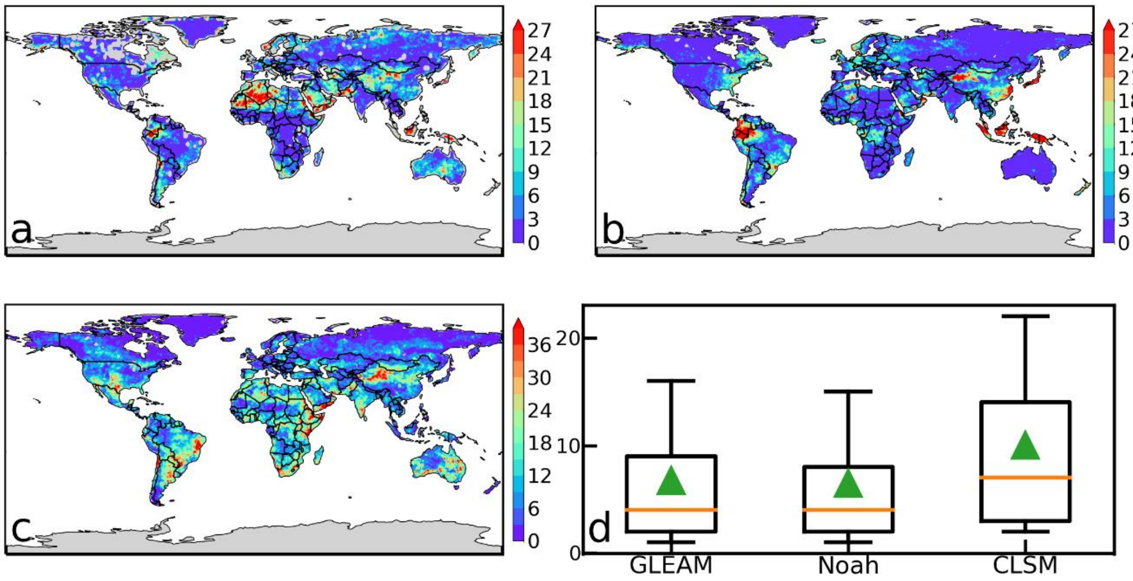

Supplementary Fig. 2 Comparison of frequencies of occurrence of flash droughts identified by GLEAM, Noah, and CLSM. a−c Spatial pattern of frequencies of occurrence of flash droughts identified by GLEAM, Noah, and CLSM. **d** Boxplots of the total number of flash droughts identified by GLEAM, Noah, and CLSM.

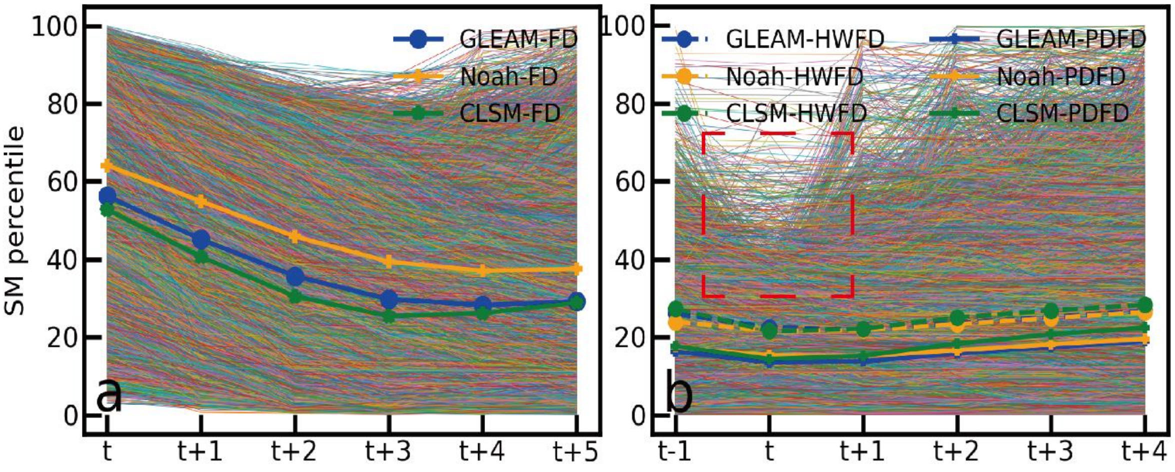

Supplementary Fig. 3 Variation of SM percentiles for flash droughts of all grid points. **a** Variation of SM percentiles for flash droughts of all grid points, identified by intensification rate. **b** Variation of SM percentiles for flash droughts of all grid points, identified by duration. The color lines represent the mean of all flash droughts detected from three models.

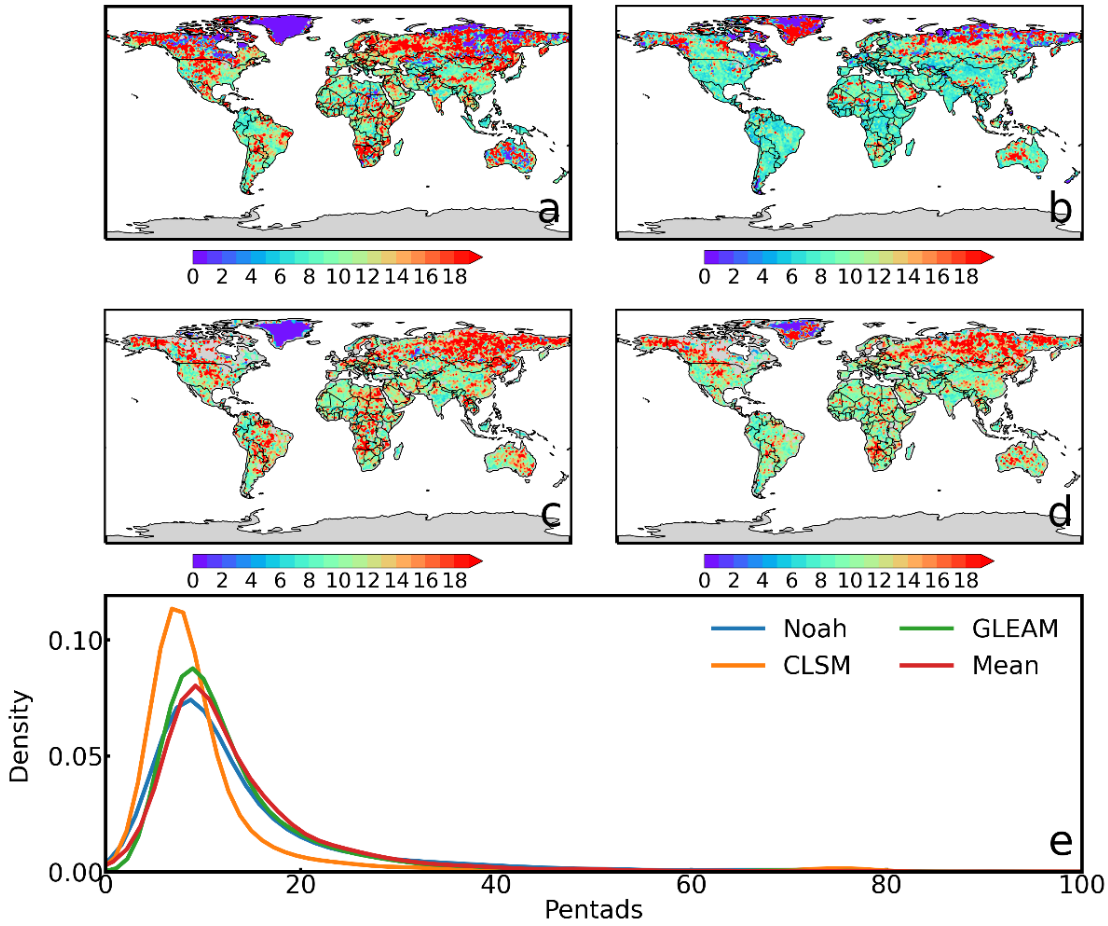

Supplementary Fig. 4 Spatial pattern of flash drought duration based on different models. a–d Mean duration of occurrence of flash droughts based on Noah, CLSM, GLEAM, and the ensemble of results from three models. **e** Probability density of the mean duration of flash droughts based on Noah, CLSM, GLEAM, and the ensemble of results from three models.

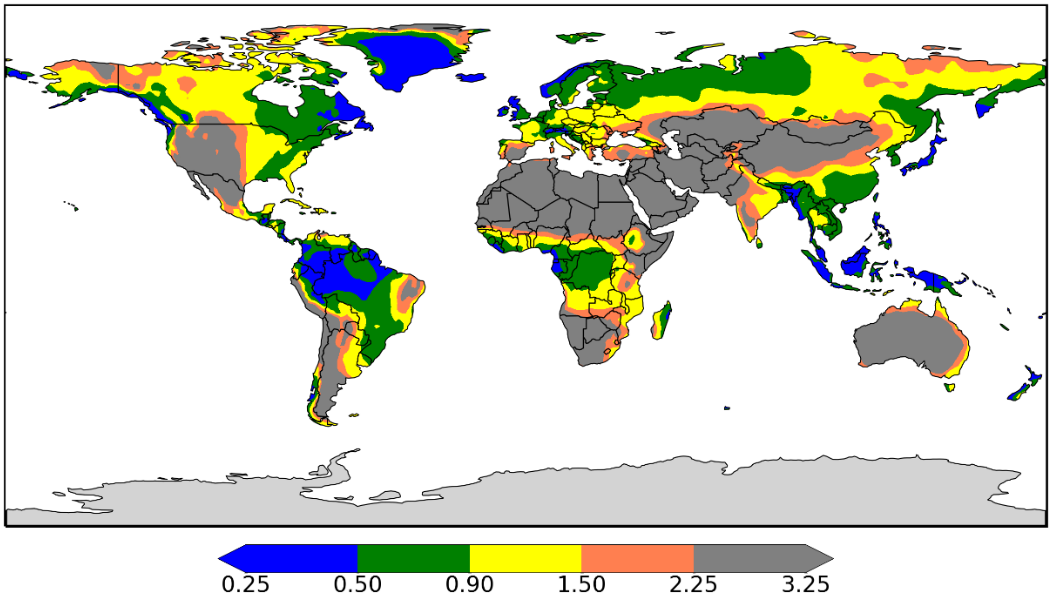

Supplementary Fig. 5 Spatial map showing the arid, transitional, and humid regions identified based on the Arid Index for the climatological period, 2000−2020.

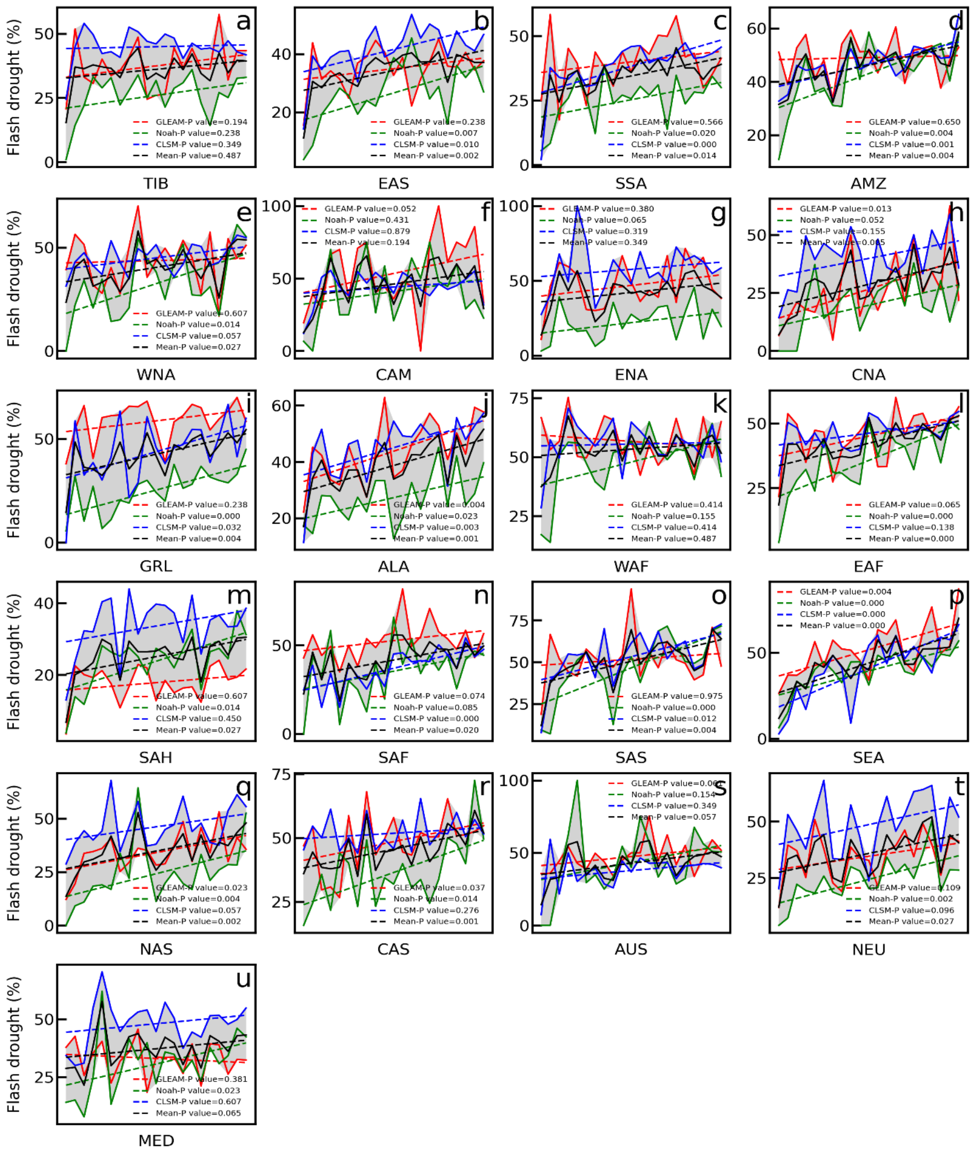

Supplementary Fig. 6 Comparison of percentages of 1-pentad onset flash droughts relative to all flash droughts for 21 regions based on GLEAM, Noah, and CLSM models. The linear annual trends in the proportion of 1-pentad onset flash droughts are estimated based on the Sen's slope estimator, and statistical significances in trends are determined based on the MK test for the entire period 2000−2020. **a**−**u** Comparison of percentages of 1-pentad onset flash droughts relative to all flash droughts for Tibet (TIB; **a**), East Asia (EAS; **b**), Southern South America (SSA; **c**), Amazon Basin (AMZ; **d**), Western North America (WNA; **e**), Central America (CAM; **f**), Eastern North America (ENA; **g**), Central North America (CNA; **h**), Greenland (GRL; **i**), Alaska (ALA; **j**), Western Africa (WAF; **k**), Eastern Africa (EAF; **l**), Sahara (SAH; **m**), Southern Africa (SAF; **n**), South Asia (SAS; **o**), Southeast Asia (SEA; **p**), North Asia (NAS; **q**), Central Asia (CAS; **r**), Australia (AUS; **s**), Northern Europe (NEU; **t**), and Mediterranean Basin (MED; **u**). Please refer to Supplementary Table 1 for more details about 21 regions.

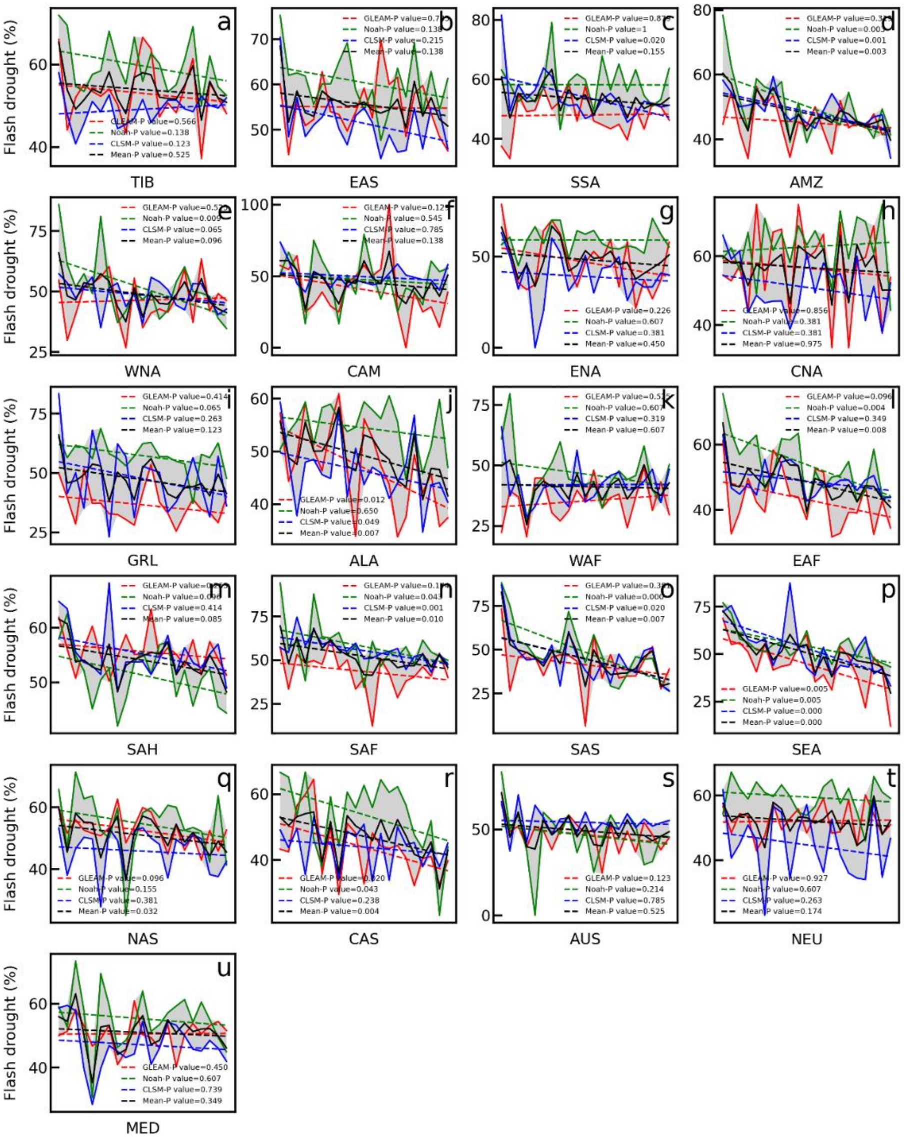

Supplementary Fig. 7 Comparison of percentages of flash droughts developing at 2-3 pentads relative to all flash droughts for 21 regions based on GLEAM, Noah, and CLSM models. The linear annual trends in the proportion of flash droughts developing at 2-3 pentads are estimated based on the Sen's slope estimator, and statistical significances in trends are determined based on the MK test for the entire period 2000−2020. **a**−**u** Comparison of percentages of flash droughts developing at 2-3 pentads relative to all flash droughts for Tibet (TIB; **a**), East Asia (EAS; **b**), Southern South America (SSA; **c**), Amazon Basin (AMZ; **d**), Western North America (WNA; **e**), Central America (CAM; **f**), Eastern North America (ENA; **g**), Central North America (CNA; **h**), Greenland (GRL; **i**), Alaska (ALA; **j**), Western Africa (WAF; **k**), Eastern Africa (EAF; **l**), Sahara (SAH; **m**), Southern Africa (SAF; **n**), South Asia (SAS; **o**), Southeast Asia (SEA; **p**), North Asia (NAS; **q**), Central Asia (CAS; **r**), Australia (AUS; **s**), Northern Europe (NEU; **t**), and Mediterranean Basin (MED; **u**). Please refer to Supplementary Table 1 for more details about 21 regions.

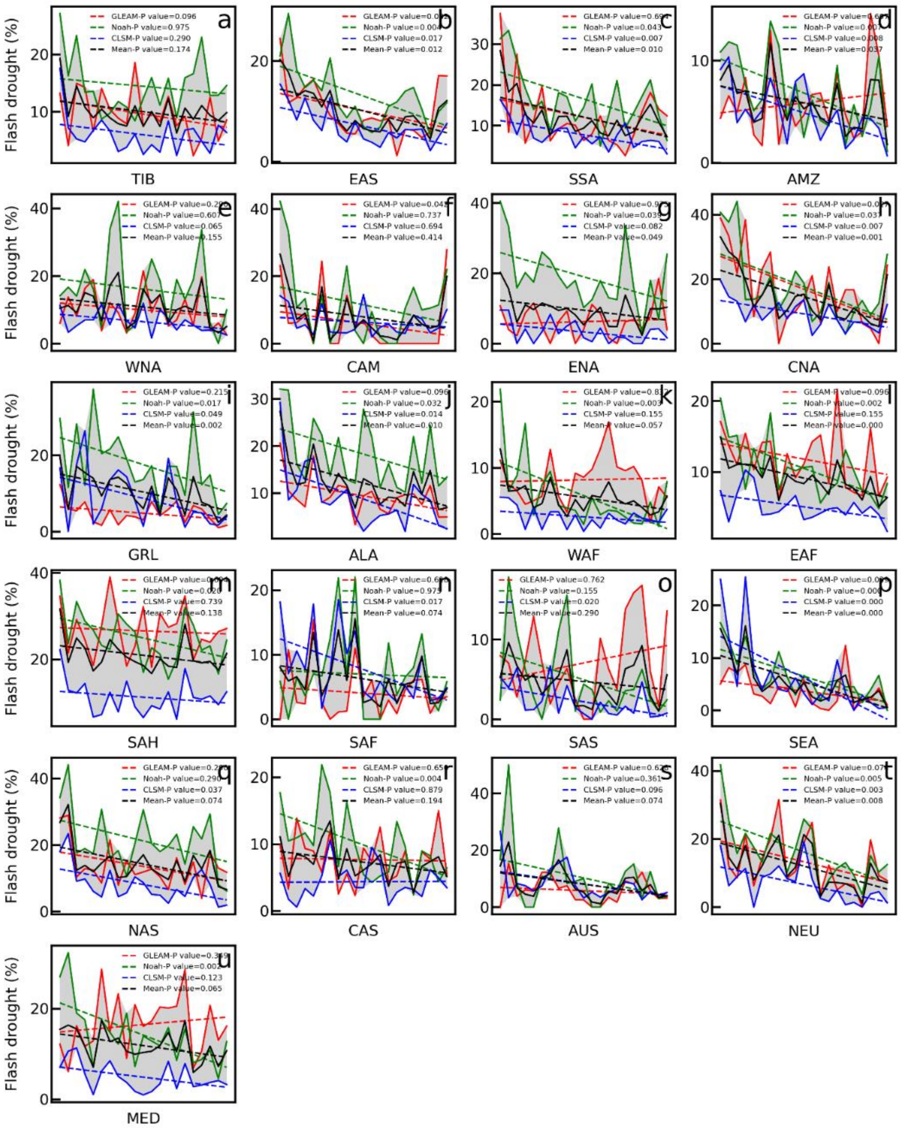

Supplementary Fig. 8 Comparison of percentages of flash droughts developing at 4-5 pentads relative to all flash droughts for 21 regions based on GLEAM, Noah, and CLSM models. The linear annual trends in the proportion of flash droughts developing at 4-5 pentads are estimated based on the Sen's slope estimator, and statistical significances in trends are determined based on the MK test for the entire period 2000−2020. **a**−**u** Comparison of percentages of flash droughts developing at 4-5 pentads relative to all flash droughts for Tibet (TIB; **a**), East Asia (EAS; **b**), Southern South America (SSA; **c**), Amazon Basin (AMZ; **d**), Western North America (WNA; **e**), Central America (CAM; **f**), Eastern North America (ENA; **g**), Central North America (CNA; **h**), Greenland (GRL; **i**), Alaska (ALA; **j**), Western Africa (WAF; **k**), Eastern Africa (EAF; **l**), Sahara (SAH; **m**), Southern Africa (SAF; **n**), South Asia (SAS; **o**), Southeast Asia (SEA; **p**), North Asia (NAS; **q**), Central Asia (CAS; **r**), Australia (AUS; **s**), Northern Europe (NEU; **t**), and Mediterranean Basin (MED; **u**). Please refer to Supplementary Table 1 for more details about 21 regions.

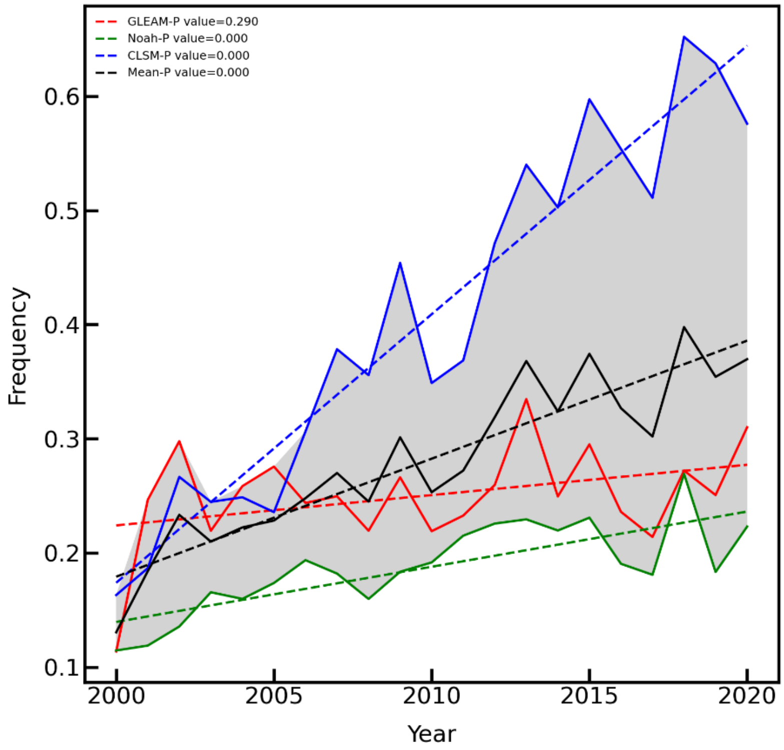

Supplementary Fig. 9 Temporal evolution of the frequency of flash droughts over the world based on GLEAM, Noah, and CLSM models. The linear annual trends in the frequency of flash droughts are estimated based on the Sen's slope estimator, and statistical significances in trends are determined based on the MK test for the entire period 2000−2020.

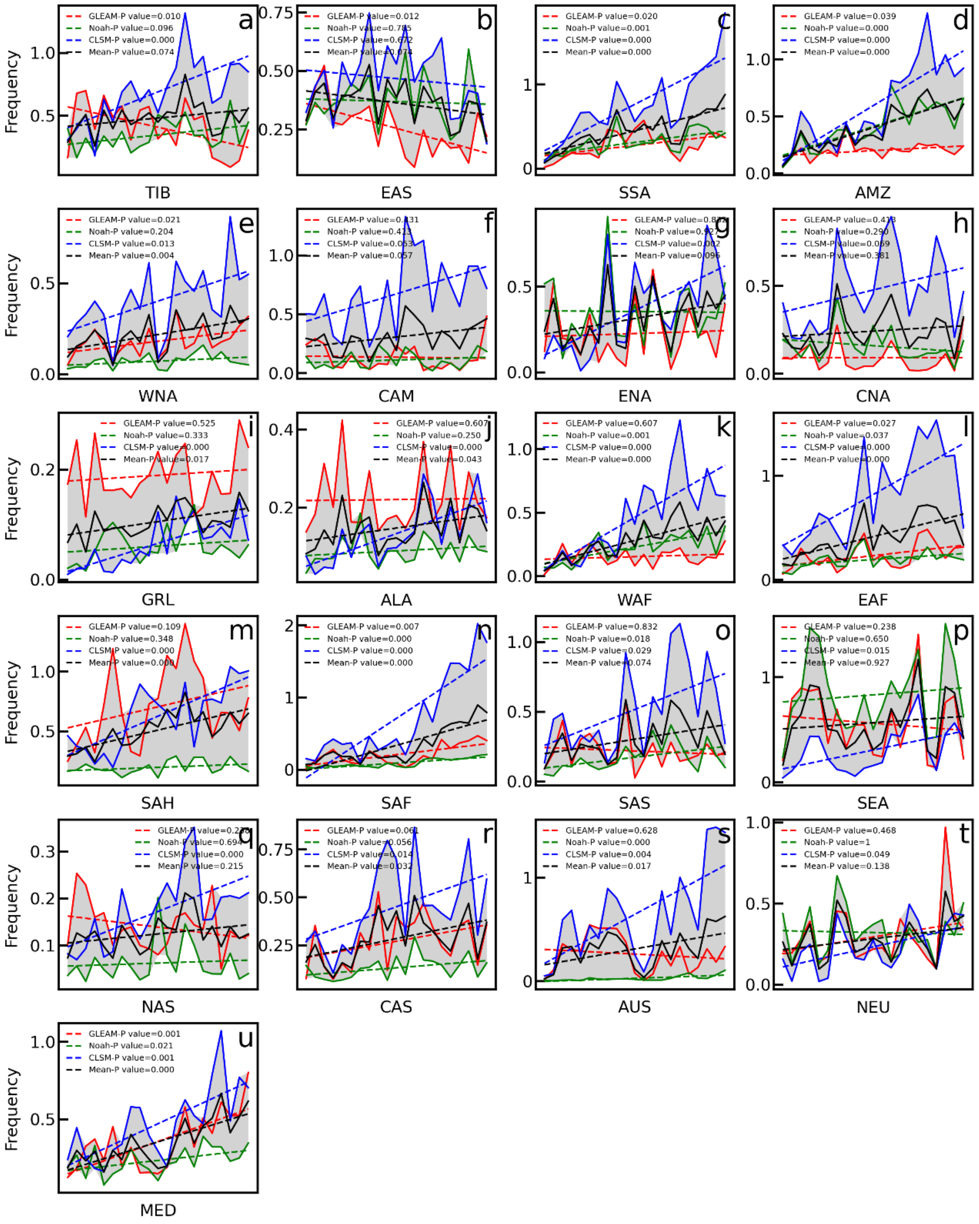

Supplementary Fig. 10 Comparison of frequency of flash droughts for 21 regions based on GLEAM, Noah, and CLSM models. The linear annual trends in the frequency of flash droughts are estimated based on the Sen's slope estimator, and statistical significances in trends are determined based on the MK test for the entire period 2000−2020. **a**−**u** Comparison of frequency of flash droughts for Tibet (TIB; **a**), East Asia (EAS; **b**), Southern South America (SSA; **c**), Amazon Basin (AMZ; **d**), Western North America (WNA; **e**), Central America (CAM; **f**), Eastern North America (ENA; **g**), Central North America (CNA; **h**), Greenland (GRL; **i**), Alaska (ALA; **j**), Western Africa (WAF; **k**), Eastern Africa (EAF; **l**), Sahara (SAH; **m**), Southern Africa (SAF; **n**), South Asia (SAS; **o**), Southeast Asia (SEA; **p**), North Asia (NAS; **q**), Central Asia (CAS; **r**), Australia (AUS; **s**), Northern Europe (NEU; **t**), and Mediterranean Basin (MED; **u**). Please refer to Supplementary Table 1 for more details about 21 regions.

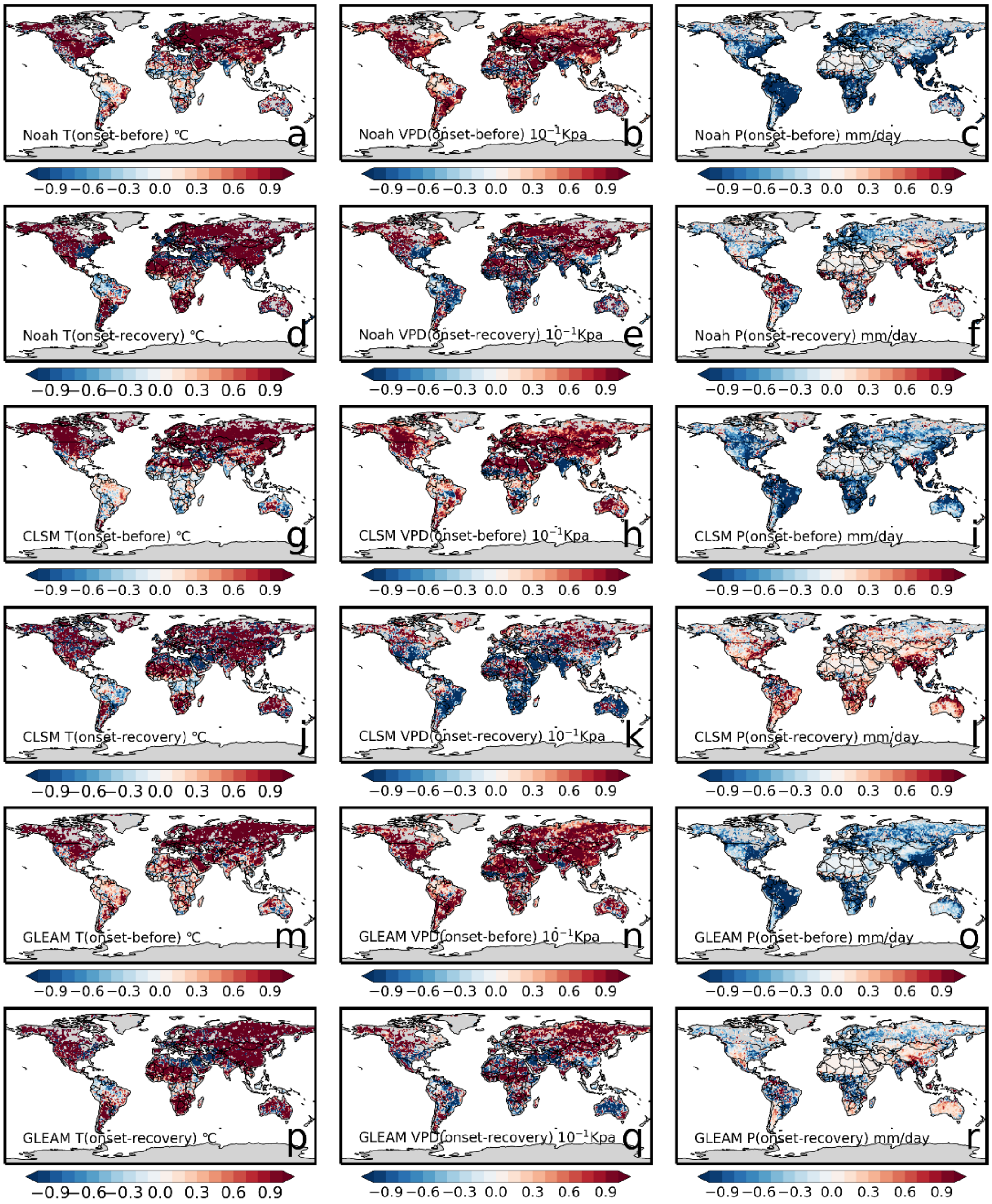

Supplementary Fig. 11 Comparison of meteorological conditions between different stages of flash droughts in light of flash droughts captured based on SM from Noah, CLSM, and GLEAM models. **a**–**c** Differences of T, VPD, and P between before (two pentads prior to flash drought) and onset stages of flash droughts according to the flash droughts captured based on SM from Noah model. **d**–**f** Differences of T, VPD, and P between onset and recovery stages of flash droughts according to the flash droughts captured based on SM from Noah model. **g**–**l** Same as **a**–**e** but for the CLSM model. **m**–**r** Same as **a**–**e** but for the GLEAM model.

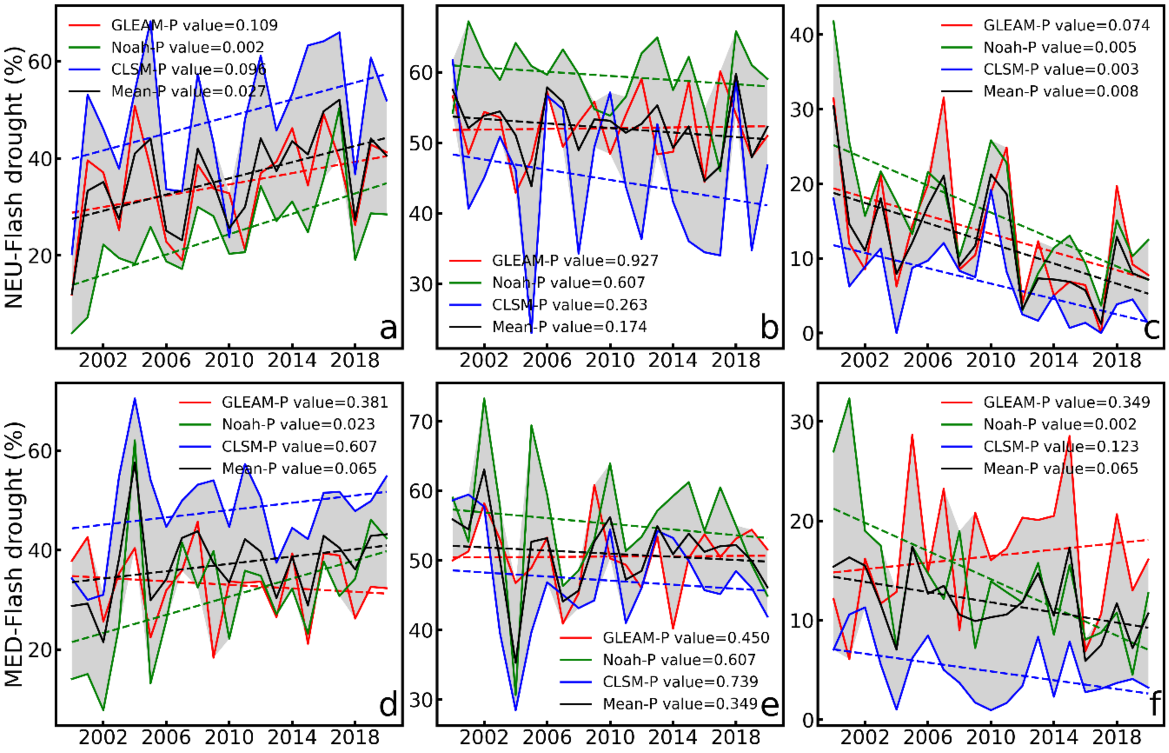

Supplementary Fig. 12 Comparison of percentages of flash droughts at different onset times relative to all flash droughts over NEU and MED based on GLEAM, Noah, and CLSM models. a−c Temporal evolutions of percentages of flash droughts with the onset times of 1-, 2-3, and 4-5 pentads relative to all flash droughts over NEU. **d−f** Temporal evolutions of percentages of flash droughts with the onset times of 1-, 2-3, and 4-5 pentads relative to all flash droughts over MED. The linear annual trends in the proportion of flash droughts at different onset times are estimated based on the Sen's slope estimator, and statistical significances in trends are determined based on the MK test for the entire study period 2000−2020.

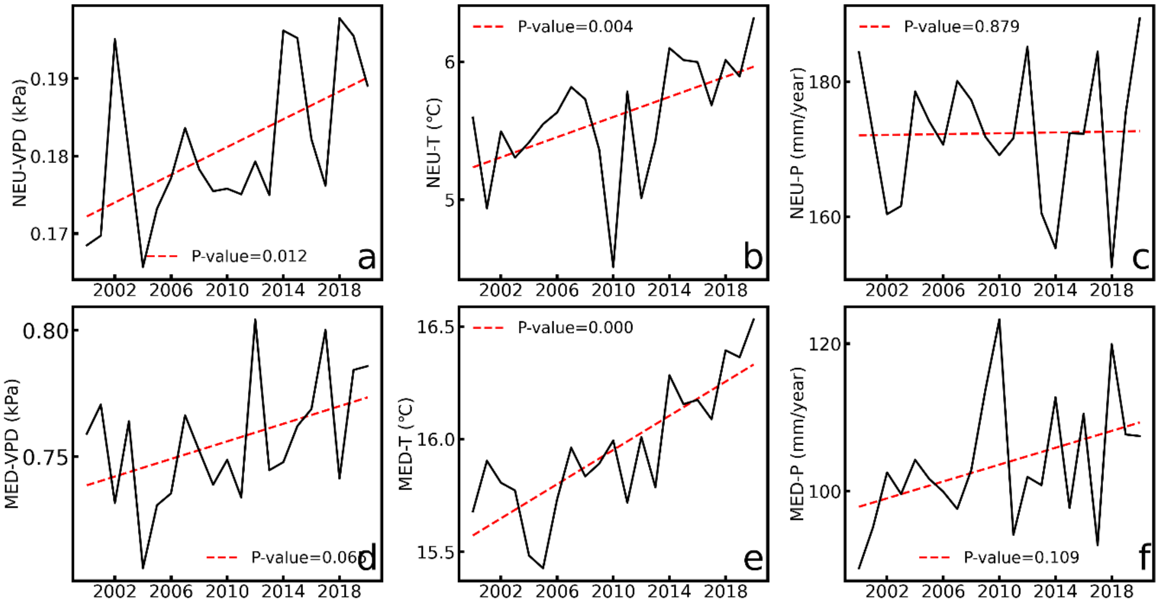

Supplementary Fig. 13 Temporal evolutions of annual mean VPD, annual mean T, and annual total P over NEU and MED. a−c Annual mean VPD, annual mean T, and annual total P over NEU. **d−f** Same as **a−b** but for MED. The linear annual trends in the annual mean VPD, annual mean T, and annual total P are estimated based on the Sen's slope estimator, and statistical significances in trends are determined based on the MK test for the entire study period 2000−2020.

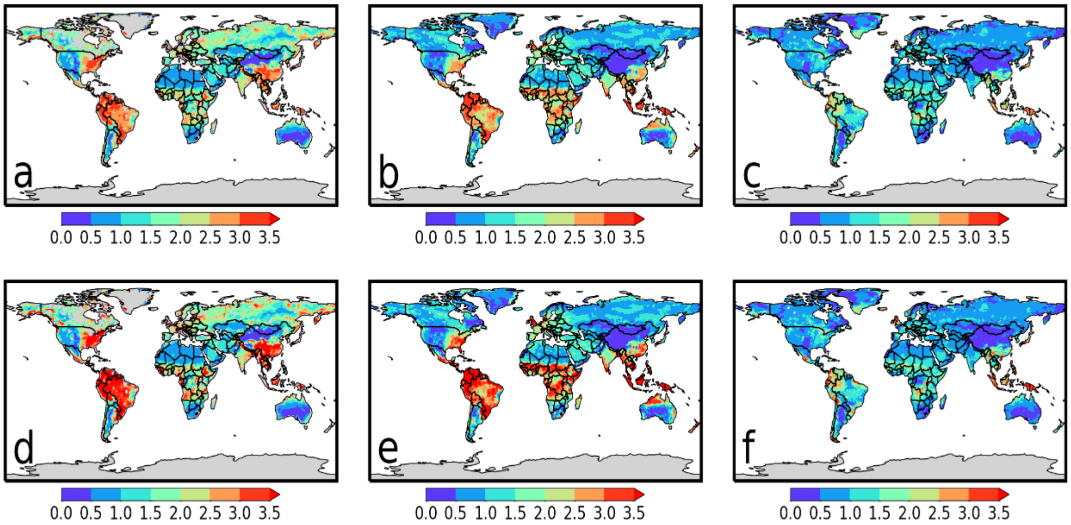

Supplementary Fig. 14 Change in concurrent extreme SM and VPD. a−b Mean PMF of concurrent extreme SM (below the 30th percentile) and VPD (above the 95th percentile) for the period 2000−2020 based on GLEAM, Noah, and CLSM datasets. **d−f** Mean PMF of concurrent extreme SM (below the 20th percentile) and VPD (above the 90th percentile) for the period 2000−2020 based on GLEAM, Noah, and CLSM datasets.

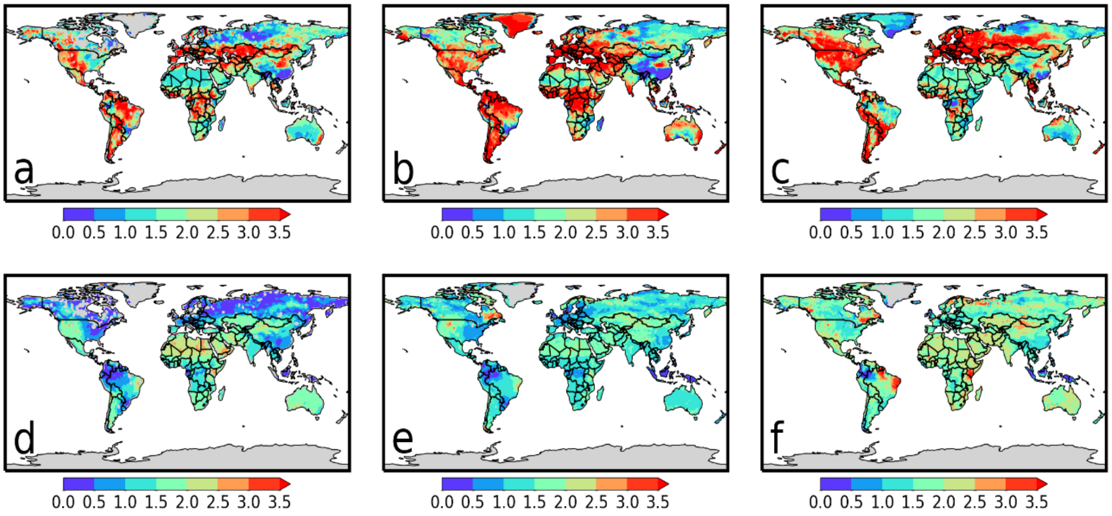

Supplementary Fig. 15 Relationship of SM-T and SM-P. a−c Mean PMF of concurrent extreme SM (below the 20th percentile) and T (above the 90th percentile) for the period 2000−2020 based on GLEAM, Noah, and CLSM datasets. **d−f** Mean PMF of concurrent extreme SM (below the 20th percentile) and P (below the 10th percentile) for the period 2000−2020 based on GLEAM, Noah, and CLSM datasets.

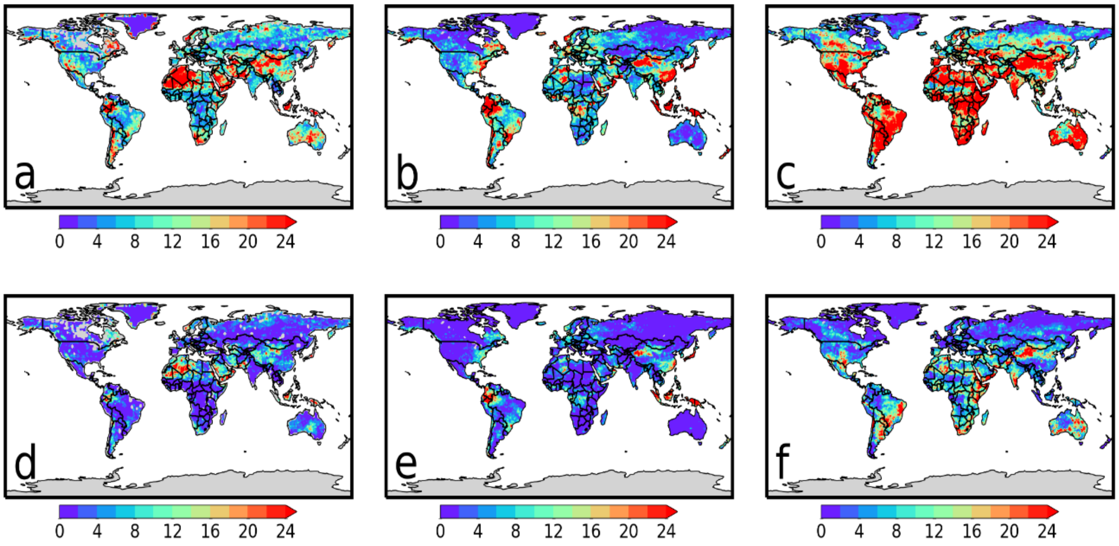

Supplementary Fig. 16 Comparison of spatial characteristics of flash droughts identified by different thresholds of SM. a−c Spatial pattern of frequencies of occurrence of flash droughts identified by the threshold of the 30th percentile SM based on GLEAM, Noah, and CLSM datasets. **d−f** Spatial pattern of frequencies of occurrence of flash droughts identified by the threshold of the 50th percentile SM based on GLEAM, Noah, and CLSM datasets.

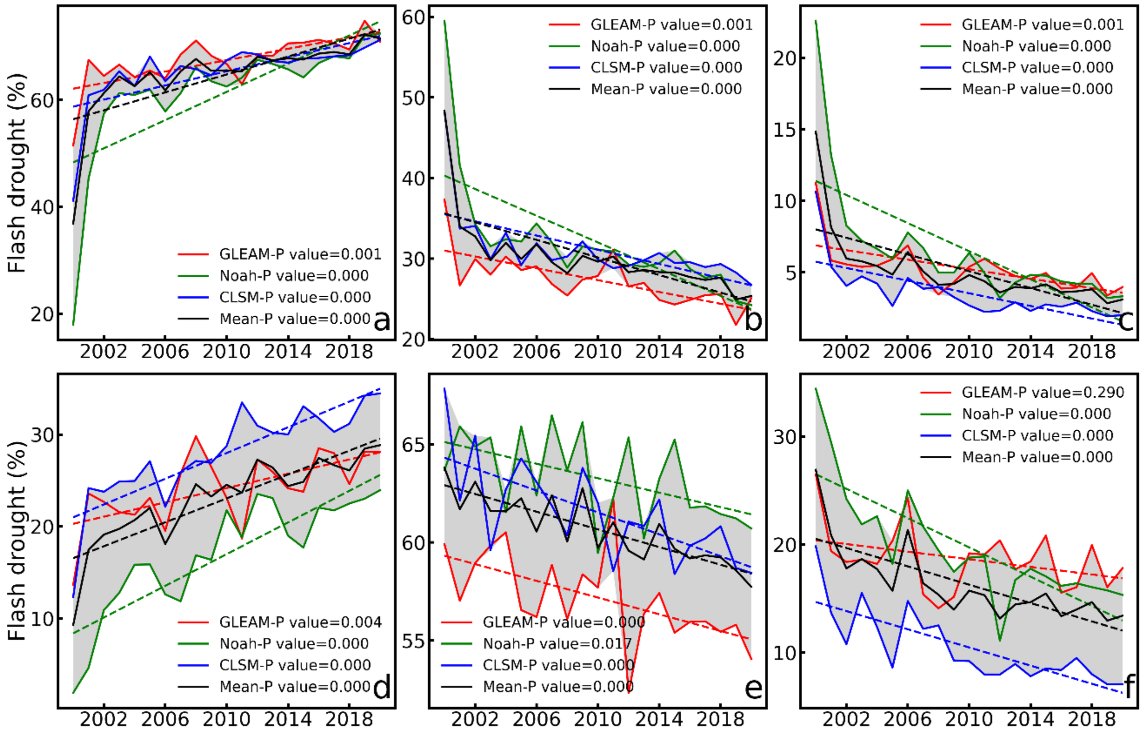

Supplementary Fig. 17 Comparison of flash droughts identified by different thresholds of SM based on GLEAM, Noah, and CLSM datasets. a–c Temporal evolutions of percentages of flash droughts identified by the threshold of the 30th percentile SM with the onset time of 1-, 2-3, and 4-5 pentads relative to all flash droughts. **d–f** Temporal evolutions of percentages of flash droughts identified by the threshold of the 50th percentile SM with the onset time of 1-, 2-3, and 4-5 pentads relative to all flash droughts.

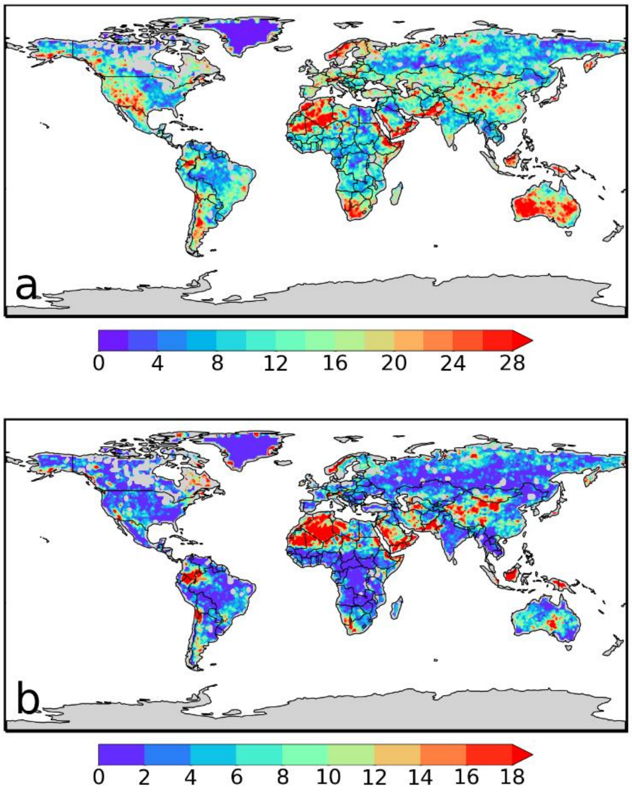

Supplementary Fig. 18 Comparison of spatial characteristics of flash droughts identified by different depths of SM. a Spatial pattern of frequencies of occurrence of flash droughts identified by the surface SM. **b** Spatial pattern of frequencies of occurrence of flash droughts identified by the root-zone SM. The root-zone and surface SM are from the GLEAM dataset.

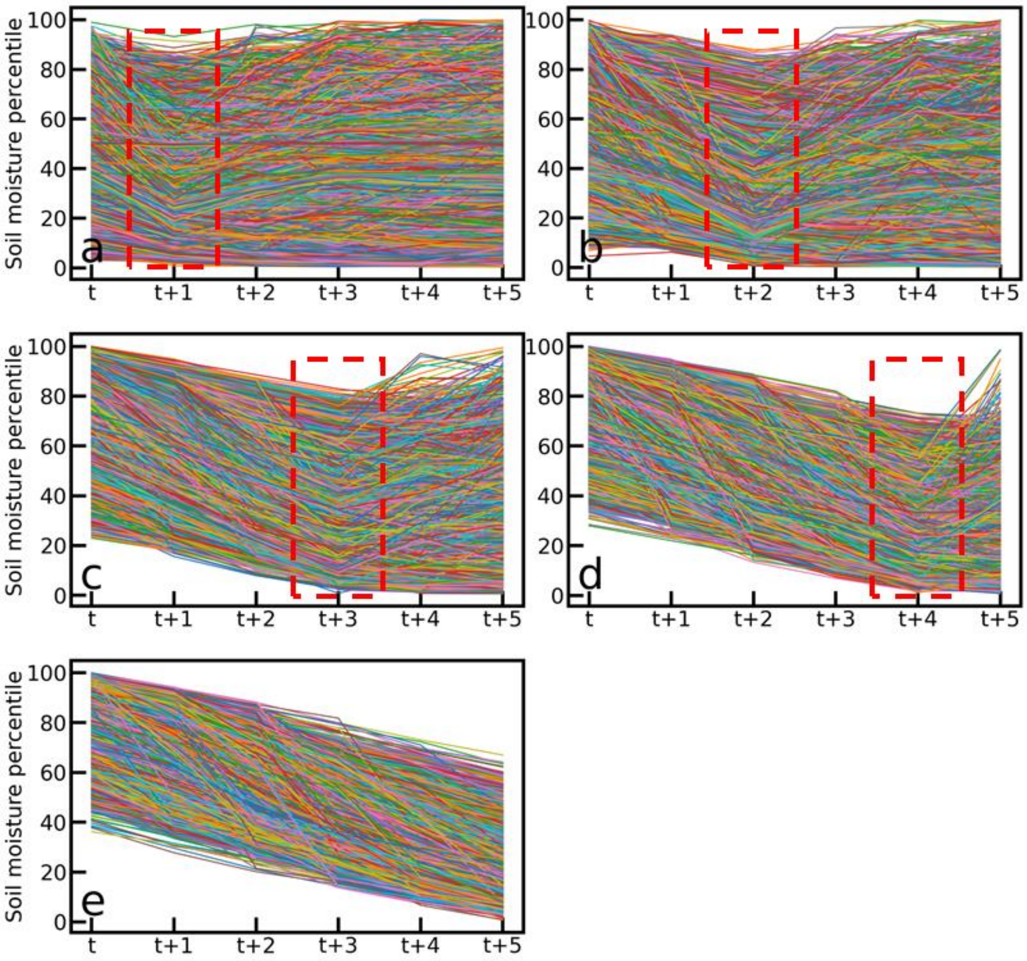

Supplementary Fig. 19 Variation in SM percentiles for each grid point of flash droughts at 1-, 2-, 3-, 4-, and 5-pentad onset times without considering the duration that can diminish crop productivity and yield (SM should not only drop below the 20th percentile but also last for at least three pentads). The red boxes highlight the events with SM decreasing to below the 40th percentile occur, but then rapidly recover up to the 40th percentile within only one or two pentads under abnormally dry conditions. **a, b, c, d, and e** correspond to the variation in SM percentiles for each grid point of flash droughts at 1-, 2-, 3-, 4-, and 5-pentad onset times, respectively.

Supplementary Table

Supplementary Table 1 List of regions used in this study (only land grid points are used in the analysis).

Name	Acronym	Latitude (°)	Longitude (°)
Australia	AUS	45S-11S	110E-155E
Amazon Basin	AMZ	20S-12N	82W-34W
Southern South America	SSA	56S-20S	76W-40W
Central America	CAM	10N-30N	116W-83W
Western North America	WNA	30N-60N	130W-103W
Central North America	CNA	30N-50N	103W-85W
Eastern North America	ENA	25N-50N	85W-60W
Alaska	ALA	60N-72N	170W-103W
Greenland	GRL	50N-85N	103W-10W
Mediterranean Basin	MED	30N-48N	10W-40E
Northern Europe	NEU	48N-75N	10W-40E
Western Africa	WAF	12S-18N	20W-22E
Eastern Africa	EAF	12S-18N	22E-52E
Southern Africa	SAF	35S-12S	10W-52E
Sahara	SAH	18N-30N	20W-65E
Southeast Asia	SEA	11S-20N	95E-155E
East Asia	EAS	20N-50N	100E-145E
South Asia	SAS	5N-30N	65E-100E
Central Asia	CAS	30N-50N	40E-75E
Tibet	TIB	30N-50N	75E-100E
North Asia	NAS	50N-70N	40E-180E
